# Percutaneous transluminal radiofrequency closure of the coronary artery in animal studies

**DOI:** 10.3892/etm.2013.1262

**Published:** 2013-08-16

**Authors:** CHENYUN ZHANG, WEI YI, YUNCHANG CAI, SHOUNIAN FANG, XINAN JIANG, ANZHI WEN, QIANG WU

**Affiliations:** Guizhou Provincial Cardiovascular Institute and Cardiology Department of Guizhou Provincial Hospital, Guiyang, Guizhou 550002, P.R. China

**Keywords:** coronary artery, radiofrequency, closure

## Abstract

The aim of this study was to investigate the safety and effectiveness of a novel method for the selective transcoronary closure of small coronary arteries by the intraluminal application of radiofrequency (RF) energy. Twenty-six small (diameter of 1–2 mm) coronary artery branches were selected in 13 dogs. An RF electrode wire (CRW-Zcy) was placed into the target vessel and a coronary balloon was used to transiently block the blood flow and limit damage to the proximal vessel. A therapeutic dosage of 20–30 W of RT energy every 10–30 sec (selected according the diameter of the target artery) was discharged via the CRW-Zcy inside a microcatheter two or three times in order to achieve arterial closure. A high dosage of 60 W every 120 sec of RF energy was used to conduct the safety study. All 26 branches were successfully closed resulting in the complete blockage of the antegrade and retrograde flows. The area of injury was limited to the target artery and the supplied myocardium. High-dose RF did not cause injury to the adjacent vessels and myocardium. The animals tolerated the procedure well without any untoward systemic effects. A follow-up angiography at two weeks revealed no evidence of recanalization or retrograde filling of the target artery. Percutaneous transluminal radiofrequency closure is a safe and effective interventional approach for closing the small coronary arteries, and is potentially valuable for further investigation.

## Introduction

The bulk of endovascular effort and innovation has been the development of devices and techniques for opening stenotic or occluded arteries. Although stenotic or occluded arteries represent the bulk of vascular diseases, there are occasions when closure or occlusion of artery is required. Examples of this include pathological coronary artery fistula (CAF) and patent ductus arteriosus (PDA). There are a numerous interventional instruments that have been used to close CAFs and PDAs, including detachable balloons, stainless steel coils, controlled-release PDA coils, the Amplatzer PDA plug, and the Jostent [polytetrafluoroethylene (PTFE)-covered stent-graft] (1–4). Each of these devices shares a common interventional theme of implanting thrombogenic or occlusive devices close the target artery. In the current study, we investigated a novel technique for the closure of coronary arteries using percutaneous transluminal coronary radiofrequency closure (PTRFC). PTRFC utilizes a modified angioplasty wire to deliver RF energy that addresses localized injury and thrombosis, and results in closure of the target artery.

## Materials and methods

### Materials

The guiding catheter (5F JL3.5, XB3.5), wire (Wizdom™, ATW™), microballoon (U-Pass, 1.5/20 mm, 2.0–2.5/10 mm to 20 mm) and microcatheter (Mass Transit, prowler 10–14) were provided by Johnson & Johnson (New Brunswick, NJ, USA). The RF electric wire (CRW-Zcy), a modified angioplasty wire and the extracorporeal RF adapter were designed and produced at the Guizhou Provincial Cardiovascular Institute and the Cardiology Department of Guizhou Provincial Hospital (Guiyang, China). The RF machine, ILEAD 2000 was acquired from Chengdu Jinjang Equipment Co., Ltd. (Chengdu, China).

### Methods

In total, 13 healthy dogs, of either gender, weighing 25–30 kg were used in this study. This study was performed in strict accordance with the recommendations in the Guide for the Care and Use of Laboratory Animals of the National Institutes of Health (5). The animal use protocol was reviewed and approved by the Institutional Animal Care and Use Committee (IACUC) of the Cardiology Department of Guizhou Provincial Hospital (Guiyang, China). The canines were anesthetized with intravenous pentobarbital (3%, 30 mg/kg), and placed onto the catheterization table in the supine position. Tracheas were intubated and artificially ventilated in order to maintain a physiological pH and pCO_2_, and pO_2_ levels. Electrodes were placed over the limbs and the infra-xiphoid area and connected to the multi-lead recorder for electrocardiogram (ECG) recordings. A plate electrode (3×5 cm) was inserted subcutaneously into the back in order to serve as an irrelevant RF electrode. Heparin (100 U/kg) was subsequently injected into the ear vein. The femoral artery was cannulated and a coronary angiogram was performed using 5F JL3.5 catheters. The target vessels for RF closure were small branches of the left anterior descending (LAD) and circumflex (Cx) arteries, 1–2 mm in diameter. The RF electric wire (CRW-Zcy, self-modified) insulated from the microcatheter was inserted into the terminal end of the target vessels via the coaxial microballoon catheter (U-Pass, 1.5/20, 2.0–2.5/10–20 mm), and 10–20 mm beyond the catheter tip. The extracorporeal end of the CRW-Zcy was connected to the RF adapters (self-modified) and the RF machine (ILEAD 2000). Once the blood flow was successfully blocked by the inflated balloon or microcatheter (Mass Transit, prowler 10–14), RF energy was discharged to the terminal segment of the vessel via the CRW-Zcy tip for the closure of the target vessel. The therapeutic dosage was 20–30 W every 10–30 sec (according to the diameter of the target artery) two or three times. Following the discharge of RF energy, a slight resistance in the movement of the wire was noted, indicating a fixed intravascular CRW-Zcy tip. Continuous and gentle pulling on the wire resulted in a sudden release from the target vessels, which freely moved the CRW-Zcy back into the microcatheter. If the proximal aspects of the target vessel were required to be closed, the same process was repeated in order to close the opening of the proximal artery branch. CRW-Zcy was subsequently removed and the microcatheter was placed back into the main coronary artery for a second angiogram 5–10 min later.

## Results

### Efficacy of target vessel closure

**Figure 1.**
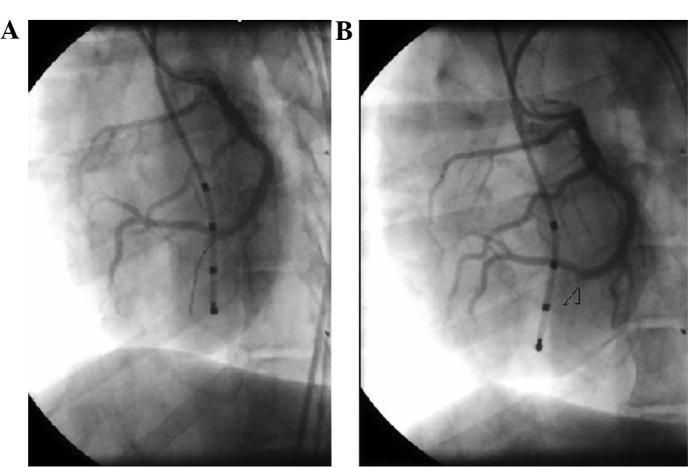
PTRFC results in complete occlusion of the target artery without residual flow. (A) PTRFC wire in place. (B) Stub-like target vessel following wire removal. PTRFC, percutaneous transluminal radiofrequency closure.

**Figure 2.**
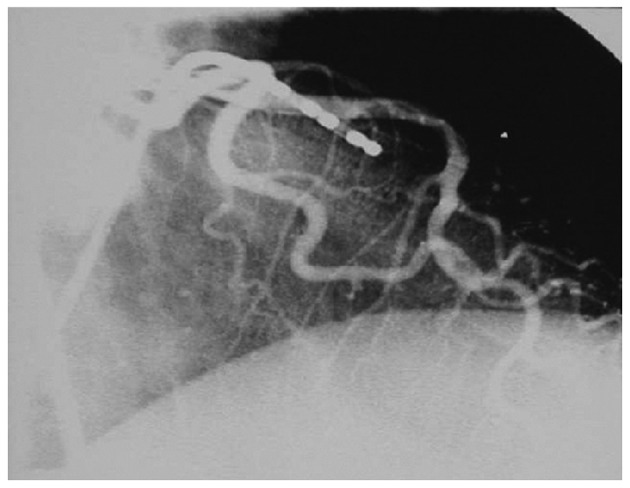
Protected proximal/middle segment of the vessel was smooth, with normal flow and no evidence of luminal strictures.

**Figure 3.**
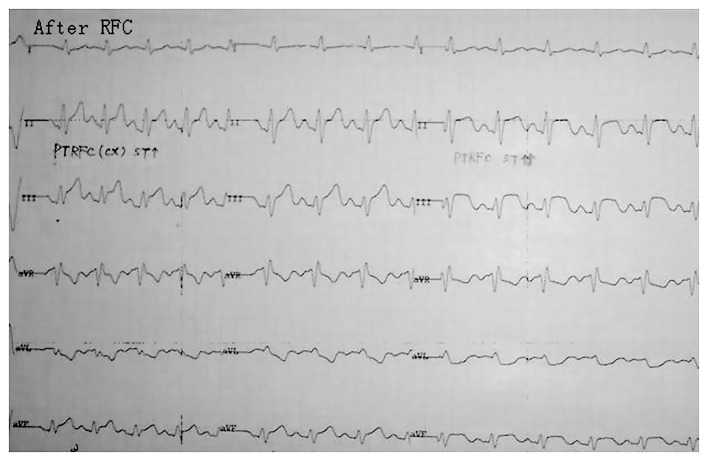
ECG at the time of PTRFC revealed an acute current injury corresponding to the myocardial area supplied by the target vessel. ECG, electrocardiogram; PTRFC, percutaneous transluminal radiofrequency closure.

**Figure 4.**
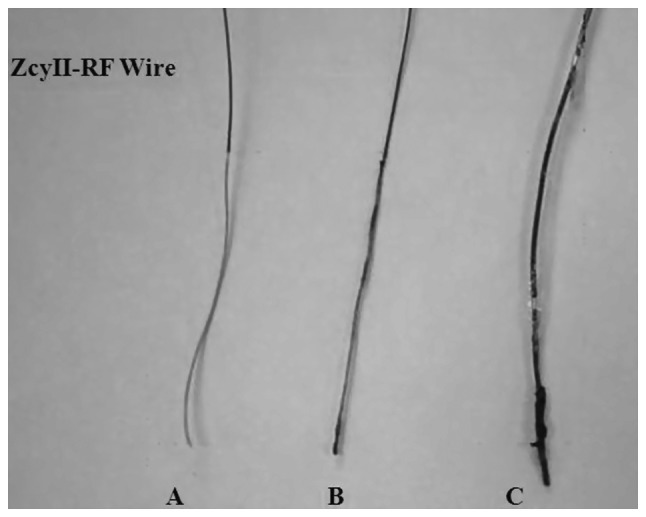
Carbonization on the tip of the wire after PTRFC. (A) New wire. Wires used with (B) a therapeutic dose and (C) a high dose. PTRFC, percutaneous transluminal radiofrequency closure.

Post-PTRFC angiograms demonstrated a complete closure in all 26 target vessels of the 13 dogs. The proximal end of the closed target vessel was small and stub-like, which was consistent with the area protected by the distal microcatheter. Immediate angiography at the time of initial closure did not reveal any evidence of residual flow (Fig. 1). The protected proximal vessel segment insulated by the CRW-Zcy from the microcatheter appeared smooth with normal flow and had no stricture of the lumen (Fig. 2). The ECG at the time of PTRFC revealed an acute current injury corresponding to the myocardial area supplied by the target vessel (Fig. 3). No evidence of diffused ischemia consistent with a large area of vasospasm or ventricular dysrhythmia was observed. Certain cases exhibited promising clinical outcomes through PTRFC. All wires were inspected following the procedure and were noted to be intact, with no evidence of structural damage. Carbonization was noted on the tips of these wires consistent with the coagulative process of PTRFC (Fig. 4).

### High-dose effect

A high current safety test was performed on all dogs. In this experiment, a single continuous RF discharge of 60 W for 120 sec was performed. There was no evidence of diffused vasospasm or cardiac arrhythmia during this experiment. Pathologic analysis revealed coagulative necrosis for 2–3 mm surrounding the target vessel and the myocardium.

### Delayed angiograms

Follow-up angiograms were performed on two dogs two weeks after the PTRFC. Revascularization was not observed.

### Pathological findings

**Figure 5.**
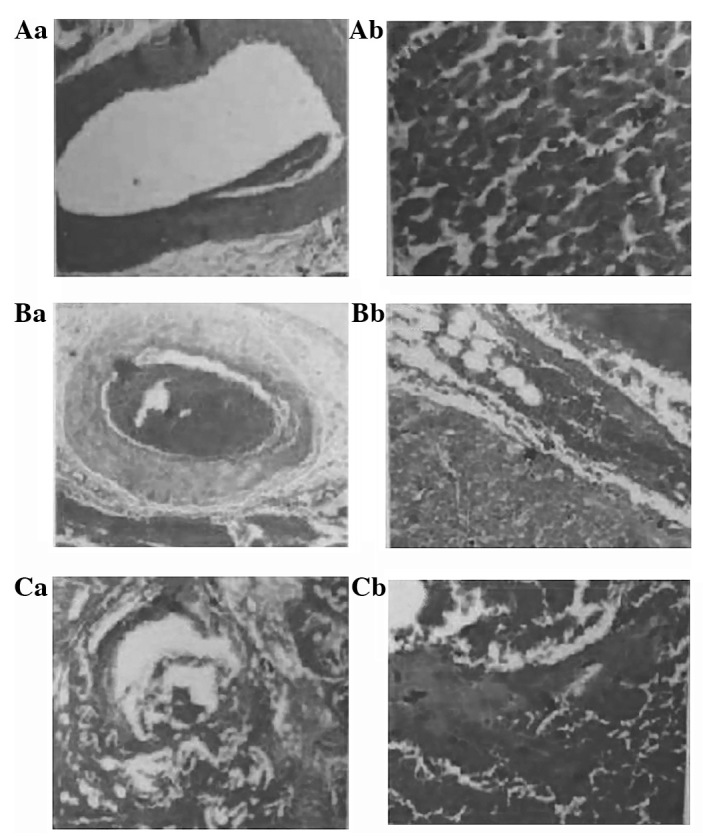
Pathological findings following percutaneous transluminal radio-frequency closure (PTRFC). Protected sections; (Aa) the blood vessel and (Ab) the myocardium are normal. Thrombus section: (Ba) mixed thrombus engorgement, blocked vessel cavities in the epicardium and myocardial layer, destroyed inner membranes of blood vessels, and atrophic smooth muscles of the middle membrane; (Bb) incomplete blockage of a few small blood vessels, swollen neighboring myocardial cells, and granular degeneration in the cell plasma. A section of the myocardium was swollen, had merged together, and resembled ‘hot, solidifying’ necrosis. Damaged section: (Ca) almost complete destruction of the vessel wall structure. (Cb) Bleeding between the neighboring myocardium, swollen myocardial cells with disappearing horizontal lines, dissolved and disappearing nuclear breaks, and myocardial necrosis.

Canine hearts were examined in five of the dogs two days following the procedure by pathological tests. In the protected section, the myocardium and blood vessels were normal (Fig. 5Aa and Ab). In the thrombus section, mixed thrombus engorgement, blocked vessel cavities in the epicardium and myocardial layer, destroyed inner membranes of blood vessels, and atrophic smooth muscles of the middle membrane were evident (Fig. 5Ba); incomplete blockage of a few small blood vessels, swollen neighboring myocardial cells and roundish red granular degeneration in the cell plasma were also observed. A section of the myocardium was swollen, had merged together, and resembled ‘hot, solidifying’ necrosis (Fig. 5Bb). In the damaged section, there was almost complete destruction of the blood vessel wall structure, an excessive quantity of red blood cells (Fig. 5Ca), bleeding between the neighboring myocardia, swollen myocardial cells with disappearing transverse lines, dissolved and disappearing nuclear breaks, and myocardial necrosis (Fig. 5Cb).

## Discussion

This study investigated the use of PTRFC to close selective coronary artery branches in dogs. The advanced techniques and instruments of percutaneous transluminal coronary angioplasty (PTCA)/RF ablation were combined to perform PTRFC in 26 selective branches of the canine LAD and Cx coronary arteries with the successful instant closure of all target vessels. Angiograms were conducted for two canines (four vessels) two weeks after the procedure and no revascularization was found. Pathological examination demonstrated that therapeutic and high doses (for the safety test) of RF discharge did not result in injury to the target vessels and the adjacent myocardium, except for closure of the selected small branches and the consequent localized myocardial infarction of the corresponding area. Limited experimental data proved that the PTRFC mechanism for closing the vessel involved thrombosis secondary to intima injury, in addition to coagulative tissue necrosis due to heated gasification by the RF current. The microcatheter insulated the RF current for protection. The introduction of CRW-Zcy in this study prevented the uninvolved vessels/myocardium from being injured when selective closure of small arteries and localized myocardial infarction were performed. Furthermore, blockage of blood flow in the target vessels by a microballoon/microcatheter reduced RF energy loss by contraflow in the target site, thus improving the efficacy of PTRFC.

The interventional process of PTRFC is valuable clinically as PTRFC may be applied for CAF closure. The presence of a pathological vessel and blood/pressure stealing (shunt from left to right) (6) leads to the occurrence of multiple serious complications with an increase in age. Complications include heart failure, myocardial ischemia, myocardial infarction, infectious endocarditis, the formation of aneurysms and ruptures, and embolisms (7–10). Therefore, symptomatic or large-shunt CAF or CAF with coronary aneurysm should be treated as quickly as possible upon diagnosis (11–16). The conventional approach of ligating or suturing by opening the chest previously had an important role in CAF treatment (11,12). Recent advances in interventional cardiology make it possible to block the fistula with various interventional instruments, such as detachable balloons, stainless steel coils, controlled-release coils, controlled-release PDA coils, Amplatzer PDA plug and Jostent (PTFE-covered stent-graft). Provided the instruments are appropriately selected, the rate of success for CAF closure is as high as 97%, and an interventional approach is predicted to replace conventional surgery as a first-choice treatment for CAF in the majority of circumstances (1–4). However, there have been occasional occurrences of embolisms (2–4), fistula dissection (3), and the dislocation of instruments (4) into the main trunk, resulting in fatalities (10). Furthermore, coronary arteries with particularly small diameters are unsuitable for closure by certain instruments, such as coils (1). To the best of our knowledge, the current study succeeded for the first time in effectively and safely closing vessels that are 1–2 mm in diameter with PTRFC, a new approach for interventional treatment. PTRFC has been applied clinically to close two CAF vessels in a 66-year-old female who had presented with angina with exertion for two years. The procedure was successful and the clinical symptoms disappeared. The ECG improved and no complications occurred. Further investigations are required in order to define the exact vessel size and indication for PTRFC, compare it with other interventional approaches, and determine its advantages and disadvantages.

Hypertrophic obstructive cardiomyopathy (HOCM) is one of the indications for PTRFC. Since Sigwart (17) treated idiopathic hypertrophic subaortic stenosis (IHSS) by percutaneous transcatheter septomyocardial ablation (PTSMA) with chemical agents in 1995, an increasing number of studies have been published regarding this technique, demonstrating satisfactory recent/mid-term outcomes and a promising future for its application (13–16,18–23). Although improvements have been made in terms of methodology, instrument quality, rate of success, mortality and degree of complication, there have been cases of severe complications, such as three branch heart block, complete atrioventricular block (19–23), pulmonary embolism (19), non-flow of the LAD artery, and ventricular fibrillation requiring conversion defibrillation (22). One of the major causes of these complications is injury of the myocardium by the uncontrollable infiltration of ethanol. However, PTRFC may prevent these complications.

RF ablation, based on endocardial mapping, is unhelpful for ventricular tachycardia (VT) when the origin is the subepicardial layer (24–26). The tree branch-like microcoronary arteries and veins distributed over the ventricular myocardium provide an anatomical basis for electrophysiological mapping and destruction of the pathological local myocardial segments. Investigations using electrophysiological mapping for VT origin via microcoronary veins followed by successful catheter RF ablation have been performed (24–26). Therefore, PTRFC may be used for treating patients with VT.

The findings in our study indicated that PTRFC is a novel interventional approach for the effective and safe closure of small pathological coronary arteries. Further investigations are required in order to define the exact indication of PTRFC, to compare its advantages and disadvantages with other interventional approaches, and to improve the different properties of the interventional instruments for clinical purposes.
